# Activation of p38 and JNK by ROS Contributes to Deoxybouvardin-Mediated Intrinsic Apoptosis in Oxaliplatin-Sensitive and -Resistant Colorectal Cancer Cells

**DOI:** 10.3390/antiox13070866

**Published:** 2024-07-19

**Authors:** Si Yeong Seo, Sang Hoon Joo, Seung-On Lee, Goo Yoon, Seung-Sik Cho, Yung Hyun Choi, Jin Woo Park, Jung-Hyun Shim

**Affiliations:** 1 Department of Biomedicine, Health & Life Convergence Sciences, BK21 Four, College of Pharmacy, Mokpo National University, Muan 58554, Republic of Korea; seosy02@mokpo.ac.kr (S.Y.S.); lso6918@mokpo.ac.kr (S.-O.L.); sscho@mokpo.ac.kr (S.-S.C.); 2 College of Pharmacy, Daegu Catholic University, Gyeongsan 38430, Republic of Korea; sjoo@cu.ac.kr; 3 Department of Pharmacy, College of Pharmacy, Mokpo National University, Muan 58554, Republic of Korea; gyoon@mokpo.ac.kr; 4 Department of Biochemistry, College of Korean Medicine, Dong-Eui University, Busan 47227, Republic of Korea; choiyh@deu.ac.kr; 5 The China-US (Henan) Hormel Cancer Institute, Zhengzhou 450008, China

**Keywords:** deoxybouvardin, reactive oxygen species, colorectal cancer, JNK, p38 MAPK, cell cycle, ROS, apoptosis

## Abstract

Colorectal cancer (CRC) remains a global health burden, accounting for almost a million deaths annually. Deoxybouvardin (DB), a non-ribosomal peptide originally isolated from *Bouvardia ternifolia*, has been reported to possess antitumor activity; however, the detailed mechanisms underlying this anticancer activity have not been elucidated. We investigated the anticancer activity of the cyclic hexapeptide, DB, in human CRC HCT116 cells. Cell viability, evaluated by MTT assay, revealed that DB suppressed the growth of both oxaliplatin (Ox)-resistant HCT116 cells (HCT116-OxR) and Ox-sensitive cells in a concentration- and time-dependent manner. Increased reactive oxygen species (ROS) generation was observed in DB-treated CRC cells, and it induced cell cycle arrest at the G2/M phase by regulating p21, p27, cyclin B1, and cdc2 levels. In addition, Western blot analysis revealed that DB activated the phosphorylation of JNK and p38 MAPK in CRC. Furthermore, mitochondrial membrane potential (MMP) was dysregulated by DB, resulting in cytochrome c release and activation of caspases. Taken together, DB exhibited anticancer activity against both Ox-sensitive and Ox-resistant CRC cells by targeting JNK and p38 MAPK, increasing cellular ROS levels, and disrupting MMP. Thus, DB is a potential therapeutic agent for the treatment of Ox-resistant CRC.

## 1. Introduction

Cancer-related mortality accounts for almost 10 million deaths annually worldwide [1]. Notably, colorectal cancer (CRC) is a global burden; CRC is the third most common cancer worldwide and accounts for almost a million deaths annually [2]. CRC progresses silently without symptoms in its early stages and tends to be in its late stages by the time it is diagnosed. Several options are available for CRC treatment; FOLFLOX, a chemotherapy based on a combination of three chemotherapeutics, fluorouracil, folinic acid, and oxaliplatin (Ox), is very effective in treating CRC as an adjuvant therapy or as a first-line treatment for advanced or metastatic CRC [3]. Ox belongs to a platinum-based chemotherapeutic agent that causes DNA cross-linking, leading to apoptosis in cancer cells [4]. However, cancer cells may utilize drug efflux mechanisms, DNA repair machinery, and detoxification pathways to combat Ox-based chemotherapy and develop resistance [5]. Targeted therapies can also be used to treat CRC. Targeting angiogenesis with bevacizumab or EGFR signaling with cetuximab may be used in combination with chemotherapy [6]. Immunotherapy such as CAR-T cell therapy is emerging as a possible alternative to chemotherapy for CRC treatment [7]. Despite the introduction of new strategies for CRC treatment, Ox remains a key chemotherapeutic agent. In line with the development of targeted therapies, it would be beneficial to identify new molecular targets to overcome the shortcomings of current treatments.

Deoxybouvardin (DB) is a non-ribosomal peptide originally isolated from *Bouvardia ternifolia,* a shrub [8]. DB was previously reported to possess antitumor activity [9]. Initially, it was considered to function as an ionophore, similar to valinomycin, or to inhibit protein synthesis by forming complexes with ribosomes [10], and it was also suggested to exert antitumor activity by modulating the level of cyclin D1 [11]. However, the detailed mechanisms underlying this anticancer activity have not yet been elucidated, and the clinical application of DB is not feasible yet.

In this study, the anticancer activity of DB was investigated in Ox-sensitive and Ox-resistant human CRC cells, HCT116, and HCT116-OxR. HCT116 served as a good model system for colorectal cancer, and HCT116-OxR allowed us to pursue Ox resistance [12]. We demonstrate that DB induces apoptosis in CRC cells by increasing reactive oxygen species (ROS) generation and activating JNK/p38 MAPK signaling. This is the first report to indicate that DB is a potential therapeutic agent for treating Ox-resistant CRC.

## 2. Materials and Methods

### 2.1. Chemical

Deoxybouvardin (purity > 95%) was obtained from the College of Pharmacy, Chungnam National University, Daejeon, Republic of Korea (Supplementary Materials Table S1).

### 2.2. Reagents

All chemicals were purchased from Sigma Aldrich (St. Louis, MO, USA) unless otherwise indicated. The culture medium was purchased from WELGENE (Gyeongsan, Gyeongsangbuk-do, Republic of Korea). Fetal bovine serum (FBS), vitamin solution, sodium pyruvate, penicillin–streptomycin (P/S), and MEM non-essential amino acid solution (NEAA) were purchased from GIBCO Invitrogen GmbH (Karlsruhe, Germany). Trypsin was purchased from HyClone (Logan, UT, USA). The phosphate-buffered saline (PBS), Tris-Glycine SDS buffer, and PRO-PREP™ Protein Extraction Solution were sourced from iNtRON Biotechnology (Seongnam, Republic of Korea). Antibodies against JNK, phosphorylated JNK (Thr183/Tyr185) (p-JNK), anti-p38, anti-phosphorylated p38 (Thr180/Tyr182) (p-p38), and anti-poly (ADP-ribose) polymerase (PARP) were purchased from Cell Signaling Technology (Danvers, MA, USA). The antibodies probing β-actin, Caspase-3, cdk inhibitors p21 and p27, cyclin B1, cdc2, Bcl-2 family proteins Bax, Bid, Bcl-2, and Bcl-xL, Apaf-1, cytochrome c (cyto c), α-tubulin, and cytochrome c oxidase subunit 4 (COX 4) were from Santa Cruz Biotechnology (Santa Cruz, CA, USA). Oxaliplatin (Ox) was purchased from MedKoo Biosciences (Durham, NC, USA).

### 2.3. Cell Culture and Treatment

The human colorectal cancer HCT116 and HaCaT cells were obtained from the American Type Culture Collection (Manassas, VA, USA). An Ox-resistant human colorectal cell line (HCT116-OxR) was obtained from the University of Texas MD Anderson Cancer Center [12]. HCT116 and HaCaT cells were maintained in RPMI-1640 and DMEM medium, respectively. Both media were supplemented with 10% FBS and 1% p/s. HCT116-OxR cells were grown in MEM supplemented with 10% FBS, 1% p/s, 1% MEM vitamin solution, 1% sodium pyruvate, 1% MEM non-essential amino acids solution, and Ox (2 μM). All cells were cultured in an incubator with 5% CO_2_ at 37 °C. The cells were then treated with DB (2, 4, or 6 nM) for 24 or 48 h. When necessary, the cells were pre-treated with the indicated concentrations of the inhibitors SP600125, SB203580, NAC, or Z-VAD-FMK for 3 h before treatment with DB (5 nM) for 48 h.

### 2.4. MTT Assay

HCT116 (5000 cells/well), HCT116-OxR (4000 cells/well), and HaCaT (8000 cells/well) cells were seeded into each well of a 96-well plate and allowed to attach for 24 h before DB treatment. The cells were then treated with DB for 24 or 48 h. After DB treatment, MTT solution was added to the wells and incubated at 37 °C for 1 h. Absorbance was recorded at 570 nm using a microplate spectrophotometer (Thermo Fisher Scientific, Vantaa, Finland). The relative cell viability in percent was obtained from triplicate experiments, and the IC_50_ values were obtained from linear interpolation.

### 2.5. Soft Agar Assay

A base layer of 0.6% agar (basal medium: Eagle’s medium, L-glutamine, gentamicin, and 10% FBS) was added to 6-well plates. The cells (8000 cells/well) were cultured in a growth medium containing 0.3% agar and the indicated concentrations of DB or Ox for 10 days. Photographs were taken under a light microscope (Leica Microsystems, Wetzlar, Germany), and the number and size of colonies were analyzed using i-Solution™ (Vancouver, BC, Canada).

### 2.6. Western Blotting

The cells were harvested and lysed using the PRO-PREP™ Protein Extraction Solution. Equal amounts of protein were electrophoresed using SDS-PAGE and transferred onto a polyvinylidene difluoride membrane. Membranes were blocked with 3% or 5% nonfat dry milk and incubated with specific primary and secondary antibodies to visualize each protein using chemiluminescence. Immunoreactive bands were quantified using ImageQuant LAS 500 (GE Healthcare, Uppsala, Sweden). ImageJ (Software version 1.4.3.67, NIH, Bethesda, MD, USA) [13] was used to measure the Western blotting, and each blot was indicated with its relative area compared to the most intense band, after being adjusted for the loading control’s intensity.

### 2.7. Cell Cycle Analysis

Adherent and floating cells were harvested, washed with PBS, and fixed in 70% ethanol at −20 °C overnight. The cells were stained with Muse™ cell cycle reagent (Luminex, Austin, TA, USA) for 30 min at room temperature. The stained cells were evaluated by a Muse™ Cell Analyzer system (Merck Millipore, Burlington, MA, USA).

### 2.8. Reactive Oxygen Species (ROS) Measurement

ROS production was measured using the Muse^TM^ Oxidative Stress Kit (Luminex). The cells were stained with Muse^TM^ Oxidative Stress Reagent working solution 30 min at 37 °C. ROS production was analyzed using the Muse^TM^ Cell Analyzer System (Merck Millipore).

### 2.9. Mitochondrial Membrane Potential (MMP) Assay

The effect of DB on MMP was determined by staining the cells with JC-1 dye (Invitrogen, Waltham, MA, USA). The mitochondrial membrane potential was monitored with a MACSQuant^®^ Analyzer 16 Flow Cytometer (Miltenyi Biotec, Bergisch Gladbach, Germany).

### 2.10. Isolation of Cell Fractions

For detection of cytochrome c, cell pellets were suspended in plasma membrane extraction buffer (10 mM HEPES (pH 8.0), 10 mM KCl, 1.5 mM MgCl_2_·6H_2_O, 1 mM EDTA, 1 mM EGTA, 0.01 mg/mL aprotinin, 0.01 mg/mL leupeptin, 0.1 mM phenylmethylsulfonyl fluoride, and 250 mM sucrose) and homogenized with 0.1% digitonin for 1 min. Cytosolic fractions were prepared by centrifugation. The pellets (mitochondrial fraction) were resuspended in a plasma membrane extraction buffer containing 0.5% Triton X-100.

### 2.11. Annexin V/7-Aminoactinomycin D (7-AAD) Staining

The Muse™ Annexin V and Dead Cell Kit (Merck Millipore) was used to detect apoptosis. The cells were resuspended in Muse™ Annexin V and Dead Cell Reagents for 20 min at RT. Stained cells were analyzed using a Muse Cell Analyzer (Merck Millipore).

### 2.12. Multi-Caspase Assay

Multi-caspase activity was determined using the Muse^TM^ Multi-caspase Kit (Luminex). The cells were then incubated with Muse^TM^ Multi-caspase Reagent at 37 °C for 30 min. Subsequently, the cells were stained with the 7-AAD working solution for 10 min prior to analysis using a Muse^TM^ Cell Analyzer system (Merck Millipore).

### 2.13. Statistical Analysis

Data are presented as means ± standard deviation (SD). One-way or two-way ANOVA was used to compare the data from several groups (GraphPad Prism 5.0; GraphPad Software; San Diego, CA, USA). Statistical significance was set at *p* < 0.05. * *p* < 0.05, ** *p* < 0.01, and *** *p* < 0.001.

## 3. Results

### 3.1. DB Inhibits the Growth of CRC Cells

**Figure 1:**
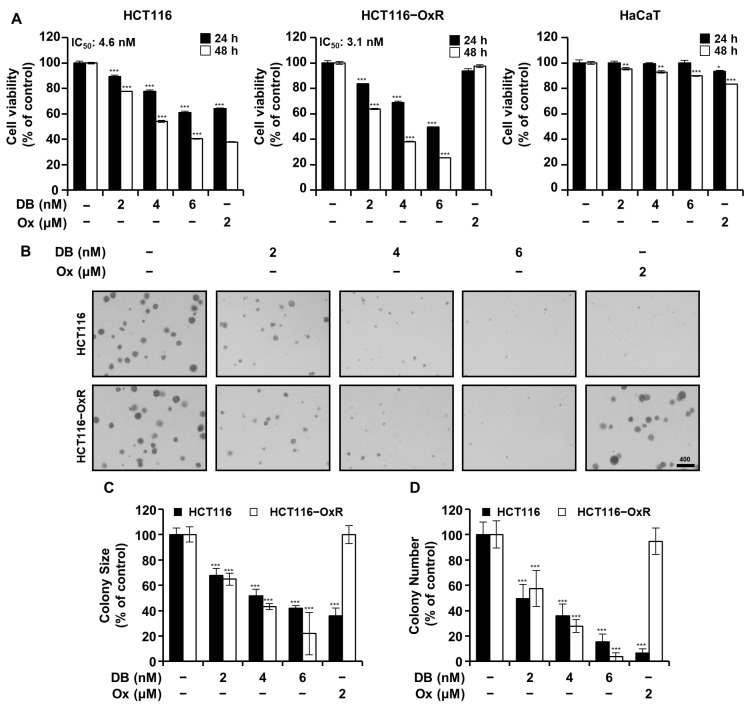
Inhibition of growth of CRC cells by DB. (**A**) Cell viability of CRC cells (HCT116 and HCT116-OxR) and HaCaT treated for 24 (black column) and 48 h (white column) with DB (0, 2, 4, and 6 nM), and Ox (2 µM) as indicated by MTT cell viability assay. Data are shown as the mean ± SD (*n* = 3). IC_50_ values for 48 h incubation. (**B**–**D**) Soft agar assay was used to determine the anchorage-independent colony growth in CRC cells (10 days incubation). (**B**) Micrograph of HCT116 and HCT116-OxR cells at 10 days after treatment. (**C**,**D**) colony size and number. * *p* < 0.05, ** *p* < 0.01, and *** *p* < 0.001 compared to vehicle only.

To determine the anti-proliferative activity of DB on CRC cells, we monitored the cell viability and proliferation of HCT116 and HCT116-OxR cells treated with DB (0, 2, 4, and 6 nM) and Ox (2 µM) using the MTT viability assay and soft agar colony formation assay. DB significantly inhibited the growth of CRC cells in dose- and time-dependent manners (Figure 1). The IC_50_ values of DB for 48 h incubation were 4.6 and 3.1 nM for HCT116 and HCT116-OxR, respectively. In the presence of Ox, the viability of HCT116 cells decreased to 37.82%, whereas that of HCT116-OxR cells was 97.53%, indicating that HCT116 cells were sensitive to Ox whereas HCT116-OxR is much more tolerant to Ox (Figure 1A). To see if the cytotoxicity of DB was cancer-specific, we treated non-cancerous human keratinocyte HaCaT cells with DB or Ox. The viability of HaCaT was slightly affected by Ox; however, it was unaffected by DB at 6 nM, with the IC_50_ value for DB appearing above 60 nM. Similar to the results of the MTT assay, the soft agar colony formation assay showed that DB inhibited colony formation in both HCT116 and HCT116-OxR cells (Figure 1B). DB treatment significantly suppressed colony formation and decreased both the size and number of colonies (Figure 1C,D). Notably, Ox (2 µM) inhibited the formation of colonies in HCT116 but not in HCT116-OxR cells. These results suggested that DB efficiently inhibited the growth of CRC cells in the nanomolar range.

### 3.2. DB Induces Apoptosis in CRC Cells by Activating JNK/p38 MAPK

**Figure 2:**
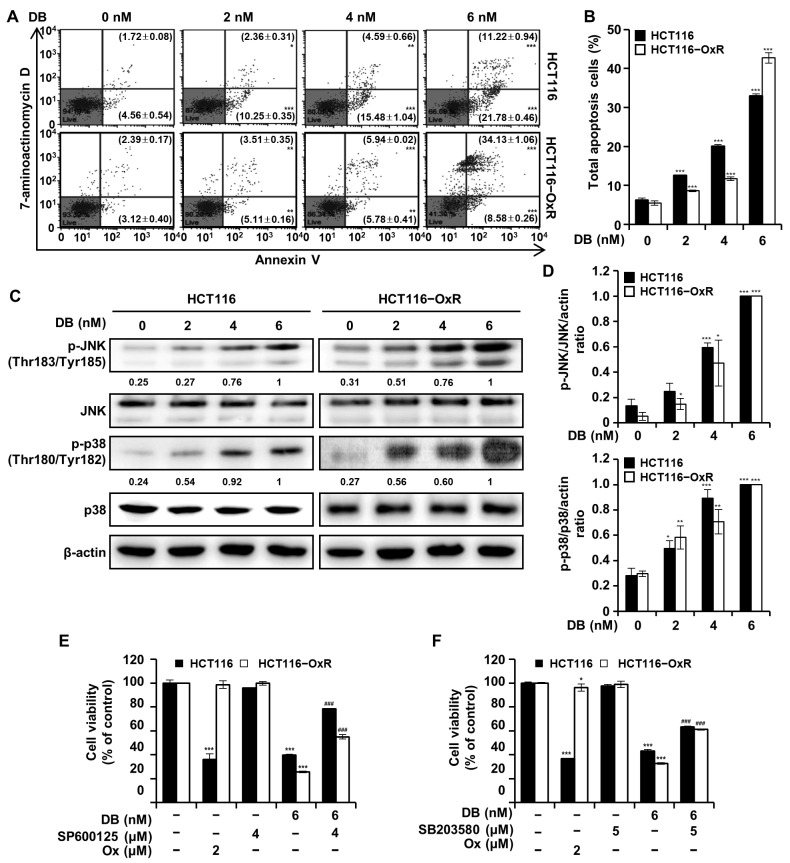
Activation of JNK and p38 MAPK in DB-induced apoptosis. CRC cells HCT116 and HCT116-OxR were analyzed by flow cytometry with annexin V/7-AAD double-staining 48 h after treatment with DB (0, 2, 4, and 6 nM). (**A**) Flow cytometry plot. (**B**) Total apoptotic cells. (**C**) Western blot analysis of cell lysates to detect p-JNK, JNK, p-p38, p38. β-actin was used as the loading control. (**D**) The ratio of phosphoprotein/total protein signal for JNK and p38. (**E**,**F**) Cell viability was assessed by the MTT assay for the CRC cells treated for 48 h with DB, SP600125, SB203580, and Ox. The data are expressed as the mean ± SD from three replicates. * *p* < 0.05, ** *p* < 0.01, and *** *p* < 0.001 compared with the control group. ### *p* < 0.001 compared with the DB-alone-treated group.

We further examined the apoptotic effects of DB on HCT116 and HCT116-OxR cells, as evidenced by the high cytotoxicity of DB in CRC cells. Flow cytometric analysis was performed on CRC cells treated with DB (0, 2, 4, and 6 nM) for 48 h after annexin V-FITC/7-AAD double-staining (Figure 2A,B). Early apoptotic cells (annexin V-FITC-positive and 7-AAD-negative) increased in the background level from 4.56% to 10.25%, 15.48%, and 21.78% by the 2, 4, and 6 nM DB treatment, respectively, in HCT116 cells. In HCT116-OxR cells, the proportion of early apoptotic cells increased from 3.12% to 5.11%, 5.78%, and 8.58%, by the 2, 4, and 6 nM DB treatment, respectively. Late apoptotic cells (annexin V-FITC-negative and 7-AAD-positive) also increased from a background level of 1.72% to 2.36%, 4.59%, and 11.22% following 2, 4, and 6 nM DB treatment, respectively, in HCT116 cells. The corresponding ratios of late apoptotic HCT116-OxR increased from 2.39% to 3.51%, 5.94%, and 34.13%, by the 2, 4, and 6 nM DB treatment, respectively. These results indicated that DB induces apoptosis in CRC cells.

The regulation of MAPK signaling is pivotal for cell survival and death. We determined whether DB modulated JNK/p38 MAPK signaling to induce apoptosis using Western blot analysis. As shown in Figure 2C,D, JNK/p38 MAPK levels were increased by the DB treatment. To determine whether the activation of JNK/p38 MAPK by DB treatment promotes apoptosis, CRC cells were pre-treated with either the JNK MAPK inhibitor, SP600125, or SB20358, an inhibitor of p38 MAPK, for 3 h before DB treatment. These pre-treatments prevented the anti-proliferative effect of DB to a certain degree (Figure 2E,F). Taken together, these results suggested that DB induces apoptosis in CRC cells by activating JNK/p38 MAPK.

### 3.3. DB Induces ROS Generation

**Figure 3:**
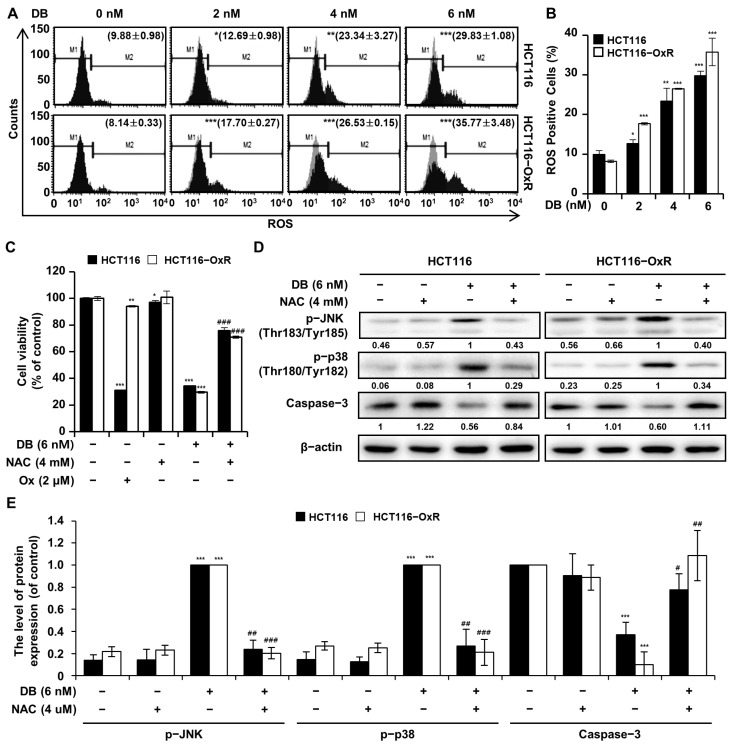
Induction of ROS by DB. CRC cells were treated for 48 h with DB, NAC, and Ox. (**A**) The cells were analyzed by flow cytometry with Muse^TM^ Oxidative Stress Kit. (**B**) The ratio of ROS-positive cells. (**C**) Cell viability was assessed using the MTT assay. (**D**) Western blot analysis to determine the levels of p-JNK, p-p38, and Caspase 3. β-actin was used as the loading control. (**E**) Graph shows the relative ratio of p-JNK, p-p38, and Casapse-3 over actin in CRC cells treated with DB or NAC. The data are shown as the mean ± standard deviation. (*n* = 3). * *p* < 0.05, ** *p* < 0.01, and *** *p* < 0.001 compared with the control group. # *p* < 0.05, ## *p* < 0.01, and ### *p* < 0.001 compared with the DB-alone-treated group.

Reactive oxygen species (ROS) generation is important for cell signaling and homeostasis; however, excessive ROS generation can result in oxidative stress and apoptosis. We examined ROS generation in the DB-treated CRC cells. As shown in Figure 3A,B, ROS generation significantly increased in HCT116 and HCT116-OxR cells treated with DB for 48 h. The cellular levels of ROS in HCT116 cells increased from 9.88% to 12.69%, 23.34%, and 29.83% with 2, 4, and 6 nM DB, respectively. The corresponding ROS levels in the HCT116-OxR increased from 8.14% to 17.7%, 26.53%, and 35.77%, by the 2, 4, and 6 nM DB treatment, respectively (Figure 3A). To determine whether increased ROS generation mediates DB-induced apoptosis, we pre-treated CRC cells with the ROS scavenger NAC (4 mM). As expected, NAC pre-treatment prevented the increase in ROS levels to some extent. The cell viability of CRC cells treated with DB was 34.39% and 29.66% for HCT116 and HCT116-OxR, respectively. The corresponding values for CRC cells pre-treated with NAC were 75.90% and 70.93% for HCT116 and HCT116-OxR cells, respectively (Figure 3C). Protein levels in CRC cells treated with DB or NAC were determined by Western blotting. NAC pre-treatment prevented the DB-induced phosphorylation of JNK and p38 (Figure 3D). The cytotoxicity of NAC pretreatment only was, if any, very little in HCT116 cells. In parallel with the phosphorylation of JNK and p38, the level of activated caspase increased upon DB treatment too. DB treatment increased the level of phosphorylated proteins JNK and p38, as well as activated caspase, whereas pretreatment with NAC prevented the increase in the level of these proteins (Figure 3E). These results suggest that DB exerted cytotoxicity in CRC cells by increasing ROS generation, which is an upstream regulator of JNK/p38 MAPK phosphorylation and caspase activation.

### 3.4. DB Induces Cell Cycle Arrest at the G2/M Phase

**Figure 4:**
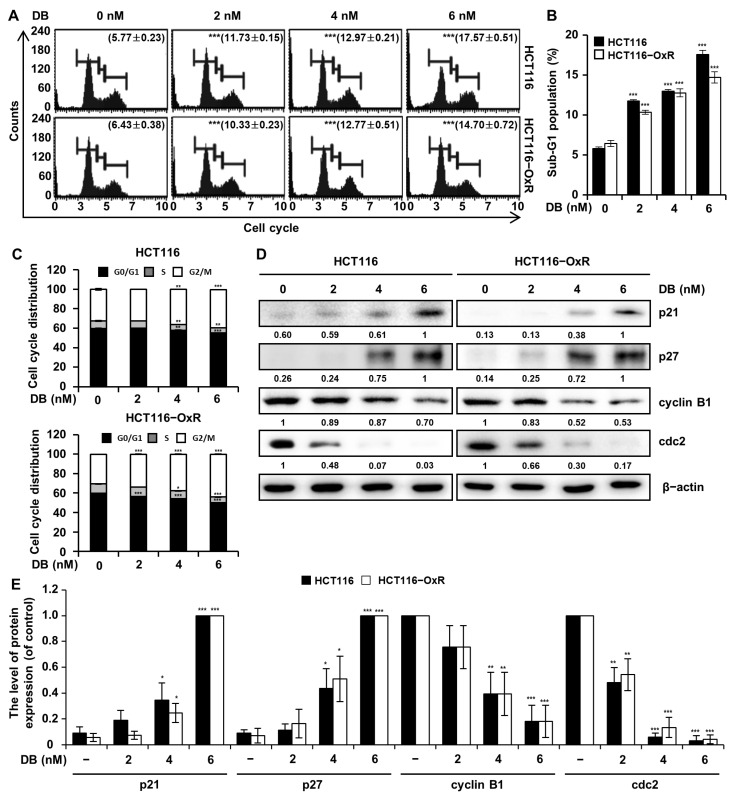
Induction of cell cycle arrest at the G2/M phase by DB. CRC cells HCT116 and HCT116-OxR were treated with DB (0, 2, 4, and 6 nM) for 48 h. (**A**) Flow cytometry analysis with PI staining. (**B**) The proportion of the cells in the Sub-G1 phase. (**C**) Cell cycle distribution. Data are shown as mean ± SD from three replicates of three independent experiments. * *p* < 0.05, ** *p* < 0.01, and *** *p* < 0.001 compared to vehicle only. (**D**) Western blot analysis of proteins related to cell cycle regulation: p21, p27, cyclin B1, and cdc2. β-actin was used as the loading control. (**E**) Graph shows the relative level of proteins p21, p27, cyclin B1, and cdc2 in CRC cells treated with DB. The data are shown as the mean ± standard deviation. (*n* = 3). * *p* < 0.05, ** *p* < 0.01, and *** *p* < 0.001 compared with the control group.

To examine the regulation of the cell cycle by DB, CRC cells treated with DB (0, 2, 4, and 6 nM) for 48 h were analyzed using flow cytometry with PI staining. As shown in Figure 4A–D, there was an increase in the proportion of cells in the G2/M phase, whereas the proportion of cells in the G1 phase decreased. The percentage of HCT116 cells in the sub-G1 phase increased from 5.77% to 11.73%, 12.97%, and 17.57%, by the 2, 4, and 6 nM DB treatment, respectively. In HCT116-OxR cells, the corresponding proportions of cells increased from 6.43% to 10.33%, 12.77%, and 14.70%, by the 2, 4, and 6 nM DB treatment, respectively. The levels of proteins involved in the cell cycle were monitored by Western blot analysis. The level of CDK inhibitors, p21 and p27, increased upon DB treatment, while the expression of cyclins, cyclin B1 and cdc2, decreased in a dose-dependent manner (Figure 4D). These results implied that DB exerted its anti-proliferative activity by inducing cell cycle arrest at the G2/M phase.

### 3.5. DB Induces Apoptosis through a Mitochondrial Pathway

**Figure 5:**
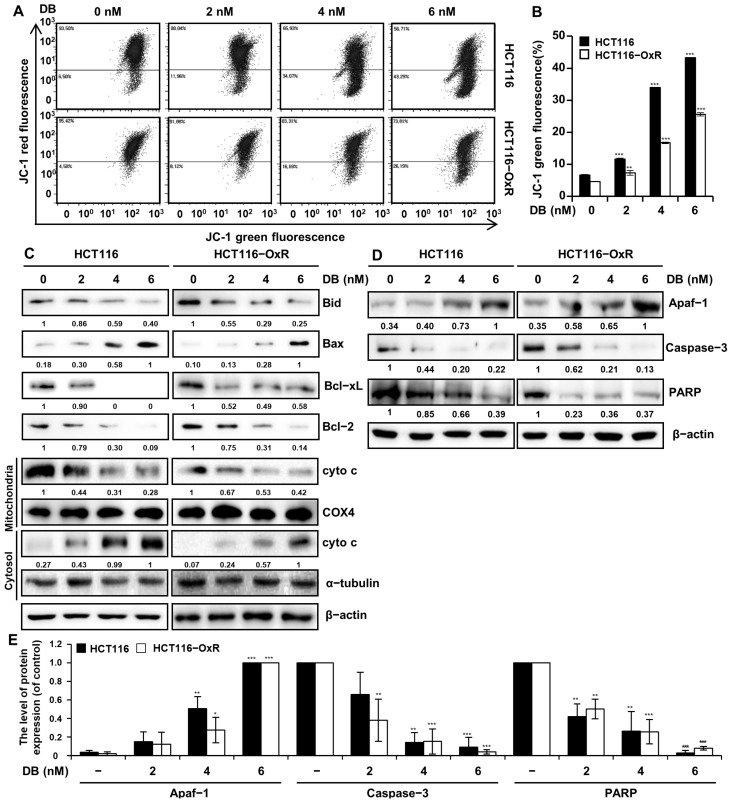
Dysregulation of mitochondrial membrane by DB. CRC cells HCT116 and HCT116-OxR were treated with DB (0, 2, 4, and 6 nM) for 48 h. (**A**) Flow cytometry analysis with JC-1 staining. (**B**) The proportion of the cells with depolarized mitochondrial membrane. Data are shown as mean ± SD of three independent experiments. ** *p* < 0.01, and *** *p* < 0.001 compared to vehicle only. (**C**) Western blot analysis of proteins regulating mitochondrial membrane permeability (Bid, Bax, Bcl-xL, and Bcl-2) and cytochrome c in mitochondrial and cytoplasmic fractions. COX4 was used as the control for mitochondrial fraction, and α-tubulin for cytoplasmic fraction. β-actin was used as the control for proteins from cell lysates. (**D**) Western blot analysis to determine the level of apoptosis-related proteins Apaf-1, Caspase-3, and PARP. β-actin was used as the control. (**E**) Graph shows the relative level of proteins Apaf-1, Caspase-3, and PARP in CRC cells treated with DB. The data are shown as the mean ± standard deviation. (*n* = 3). * *p* < 0.05, ** *p* < 0.01, and *** *p* < 0.001 compared with the control group.

Disruption of the MMP is an indicator of apoptosis. To verify whether DB-induced apoptosis was related to mitochondrial dysfunction, flow cytometry analysis was performed on CRC cells treated with DB after JC-1 staining (Figure 5A,B). The green fluorescence of JC-1, an indicator of mitochondrial damage, was increased in CRC cells following DB treatment. In contrast, the red fluorescence of JC-1, an indicator of functionally healthy mitochondria, decreased. The proportion of HCT116 cells with mitochondrial dysfunction increased from the background level of 6.64% to 11.67%, 33.95%, and 43.31% after treatment with 2, 4, and 6 nM DB, respectively. The proportion of HCT116-OxR cells increased from 4.63% to 7.28%, 16.68%, and 25.59%, by the 2, 4, and 6 nM DB treatment, respectively. The release of mitochondrial cytochrome c into the cytoplasm was analyzed by Western blotting of both mitochondrial and cytoplasmic fractions of CRC cells. As shown in Figure 5C, DB treatment decreased the levels of mitochondrial cytochrome c and increased cytoplasmic cytochrome c. In addition, Western blot analysis was performed to determine the levels of Bcl-2 family proteins and apoptosis-related proteins. Notably, DB increased the levels of the pro-apoptotic Bcl-2 family proteins Bax as well as Apaf-1. In contrast, there was a decrease in the levels of the anti-apoptotic Bcl-2 family proteins Bid, Bcl-2, and Bcl-xL, as well as Caspase-3 and PARP.

### 3.6. DB Induces Apoptosis by Mediating Caspase Activation

**Figure 6:**
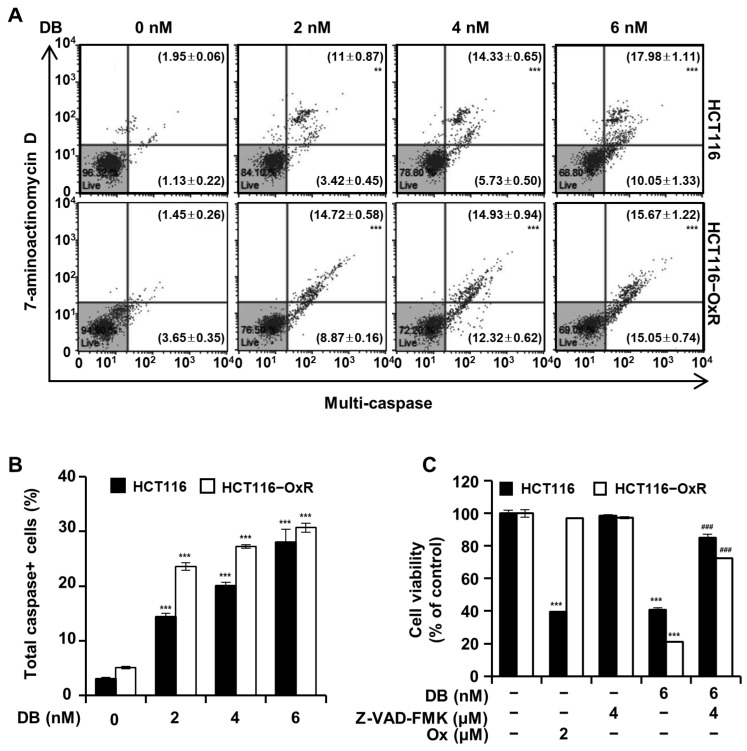
Caspase activation by DB. CRC cells HCT116 and HCT116-OxR were treated with DB (0, 2, 4, and 6 nM) for 48 h. (**A**) Flow cytometry analysis with a Muse^®^ Multi-caspase Kit. (**B**) The proportion of the cells with activated caspases was obtained by adding upper right (caspase+ and dead cells) and lower right (caspase+ and live cells). (**C**) Cell viability assessed by the MTT assay for the CRC cells treated for 48 h with DB, Z-VAD-FMK, and Ox as indicated. Data are presented as mean of three replicates. ** *p* < 0.01 and *** *p* < 0.001 compared to vehicle only. ### *p* < 0.001 compared with the DB-alone-treated group.

To examine the activation of caspases following DB treatment, flow cytometry was performed using the Muse^TM^ Multi-caspase Kit. Flow cytometry revealed that DB upregulated the activation of multiple caspases (Figure 6). The proportion of HCT116 cells with activated caspases, upper right (caspase+ and dead cells) and lower right (caspase+ and live cells), increased from the background level of 3.08% to 14.42%, 20.07%, and 28.03% by the 2, 4, and 6 nM DB treatment, respectively. In HCT116-OxR cells, these proportions increased from 5.10% to 23.58%, 27.25%, and 30.72% by the 2, 4, and 6 nM DB treatment, respectively. We hypothesized that the activation of caspase mediated the DB-induced apoptosis. To see if the activation of caspase is crucial in the progression of DB-induced apoptosis, cells were treated with co-administration of DB and Z-VAD-FMK, a pan-caspase inhibitor. The viability of CRC cells treated with DB only was 40.83% and 21.17% for HCT116 and HCT116-OxR cells, respectively. In contrast, the corresponding vitality when DB and Z-VAD-FMK were combined was 84.96% and 72.37%, respectively (Figure 6C). These results demonstrate that DB-induced apoptosis of CRC cells is mediated by caspase activation.

## 4. Discussion

In addition to their effectiveness in suppressing cancer cell growth, the side effects of chemotherapeutics are a concern. Therefore, the side effects of the new chemotherapy regimens may hamper the use of anticancer drugs [14]. Ox is a third-generation platinum-based chemotherapeutic agent with great improvement in neurotoxicity and nephrotoxicity that exists in early platinum-based anticancer drugs, such as cisplatin and carboplatin [4]. Moreover, Ox has demonstrated anticancer activity in several cancers and is considered a first-line drug for treating colorectal, gastric, and pancreatic cancers. The combination of Ox, folinic acid, and 5-fluorouracil, known as the FOLFOX regimen, is very effective in treating CRC [3]. The emergence of new chemotherapies to treat cancer and the development of drug resistance have been repeatedly reported. Although the current regimen of FOLFOX is quite effective, resistance is developed, and overcoming Ox resistance is very important in treating CRC.

In this study, we observed that DB has anti-proliferative activity on human CRC HCT116 and HCT116-OxR cells but not on human epidermal keratinocyte HaCaT cells (Figure 1). This indicates that DB could be a selective chemotherapeutic agent applicable to both Ox-sensitive and Ox-resistant CRC. Apoptosis is an attractive target in cancer therapy as it is characterized by the efficient removal of cellular contents without damaging the surrounding tissues. Annexin V/7-AAD double-staining flow cytometry revealed that DB treatment induced apoptosis in both HCT116 and HCT116-OxR cells (Figure 2A,B). To further investigate apoptosis induced by DB treatment, we evaluated the effect of DB treatment on JNK and p38 MAPK signaling. DB treatment increased the phosphorylation of JNK and p38 MAPK (Figure 2C,D). Moreover, SP600125, an inhibitor of p38, and SB203580, an inhibitor of JNK, effectively prevented DB cytotoxicity (Figure 2E,F). It has been speculated that JNK and p38 MAPK signaling modulate the expression and post-translational modification of several proteins involved in apoptosis [15,16]. It would be interesting to elucidate how JNK and p38 MAPK mediate apoptosis in CRC cells.

ROS generation is involved in various physiological and pathophysiological processes in cancer cells [17]. The basal ROS level in cancer cells is typically higher than that in normal cells [18]. However, excessive amounts of ROS may result in cytotoxicity and lead to apoptosis of cancer cells [19]. We observed increased cellular ROS levels in CRC cells treated with DB (Figure 3A,B). In contrast, pre-treatment with NAC (4 mM) prevented the induction of apoptosis by DB, indicating that ROS generation mediates the apoptotic process. Notably, the phosphorylation of JNK and p38 MAPK was not inhibited by NAC pre-treatment. This illustrates that the phosphorylation of these kinases precedes the increase in ROS generation (Figure 3C).

Cytotoxicity of anticancer therapeutics may involve cell cycle regulation. For example, the anticancer activity of Ox is related to cell cycle arrest during the G2/M transition [20]. Similarly, DB induced the accumulation of cells in the G2/M transition (Figure 4). Formation of the cyclin B1/cdc2 complex is important for cell cycle progression in the G2/M phase [21], and our results indicated that the cyclin B1/cdc2 complex was downregulated by DB treatment. Additionally, we found that an increase in the level of p21 and p27 inhibited the function of cyclin B1/cdc2 [22]. Both p21 and p27, belonging to the CDK-interacting protein/kinase inhibitory protein, are associated with susceptibility of cancer cells to anticancer drugs [23].

ROS generation above the threshold of cellular capacity leads to depolarization of the mitochondrial membrane [24] and apoptosis [25]. Furthermore, a shift was noticed in the balance between pro- and anti-apoptotic Bcl-2 family proteins [26]; the levels of the anti-apoptotic proteins Bcl-2 and Bcl-xL decreased, whereas that of the pro-apoptotic protein Bax increased and the shift in the balance of Bcl-2 family proteins resulted in the release of cytochrome c from the mitochondria into the cytoplasm (Figure 5C). The decrease in the level of pro-apoptotic Bid is an indication of the activation by cleavage [27]. The elevation of Apaf-1 levels and cleavage of procaspase-3 and PARP indicated that DB-induced apoptosis was mediated by the intrinsic apoptotic pathway (Figure 5D). Caspase activation is a key process in apoptosis [28]. We assessed the activation of caspases by a multi-caspase assay and found that DB induced the activation of multiple caspases (Figure 6A,B). This finding implies that DB-induced apoptosis is involved in caspase activation. The treatment of CRC cells with Z-VAD-FMK, a pan-caspase inhibitor, partially prevented DB-induced apoptosis (Figure 6C). This indicated that DB-induced apoptosis was mediated by caspase activation.

While we observed that DB is effective in treating oxaliplatin-resistant HCT116 cells, the result is limited to HCT116 cells. Further study will reveal if these results are applicable in other colorectal cancer cells and even other types of cancer cells. The generation of ROS, which could be harmful to surrounding tissue, will have to be measured in a mouse model or other studies. In addition, more advanced research models including 3-D cell culture models and xenograft models would enlighten our understanding of antitumor activity of DB. Up to now, there have been no reports suggesting the genotoxicity or cytotoxicity of deoxybouvardin and bouvardin.

In conclusion, we demonstrate that DB inhibits the growth of both Ox-sensitive and Ox-resistant CRC HCT116 and HCT116-OxR cells. DB induces ROS generation, cell cycle arrest at the G2/M phase, activation of JNK and /38 MAPK, depolarization of MMP, and activation of caspases. Our results support the further development of DB as a chemotherapeutic agent for treating CRC.

## Data Availability

Data are contained within the article or Supplementary Materials.

## Supplementary Materials

The following supporting information can be downloaded at: https://www.mdpi.com/article/10.3390/antiox13070866/s1. Table S1: Deoxybouvardin: white amorphous powder; ESI-MS, *m*/*z* 757.3 [M + H]^+^, *m*/*z* 797.3 [M + Na]^+^, *m*/*z* 755.2 [M − H]^−^; ^1^H and ^13^C NMR data.
