# Supplementary material for: Activation of p38 and JNK by ROS Contributes to Deoxybouvardin-Mediated Intrinsic Apoptosis in Oxaliplatin-Sensitive and -Resistant Colorectal Cancer Cells
Source: Antioxidants (Basel). 2024 Jul 19;13(7):866. doi: 10.3390/antiox13070866 (PMC11273932; doi:10.3390/antiox13070866)
Supplement: Supplementary file 1 [file antioxidants-13-00866-s001.zip › antioxidants-3061808-supplementary.pdf]

Table S1. ^{1}H NMR and ^{13}C NMR spectroscopy data of deoxybouvardin (in $CDCl_3$ 300 and 75 MHz).

Position		^{1}H	^{13}C
Ala1	α	4.78 (m)	44.6
	β	1.33 (d, *J* = 6.7 Hz)	20.8
	C=O		172.4
Ala2	α	4.45 (m)	47.7
	β	1.27 (d, *J* = 6.8 Hz)	16.5
	C=O		172.7
Tyr3	α	3.62 (dd, *J* = 9.8, 5.7 Hz)	68.3
	β	3.33 (m)	32.6
	γ		130.6
	δ*2	7.01 (ov)	130.3
	ε*2	6.81 (ov)	114.1
	ζ		158.4
	C=O		170.7
	NMe	2.83 (s)	39.9
	OMe	3.79 (s)	55.3
Ala4	α	4.81 (m)	46.4
	β	1.05 (d, *J* = 6.5 Hz)	18.5
	C=O		171.7
Tyr5	α	5.39 (d, *J* = 9.8 Hz)	54.4
	βa	3.66 (m)	36.9
	βb	2.62 (d, *J* = 9.8 Hz)	
	γ		135.6
	δa	7.24 (d, *J* = 8.5 Hz)	131.1
	δb	7.42 (d, *J* = 8.5 Hz)	133.0
	εa	6.83 (ov)	124.2
	εb	6.50 (d, *J* = 8.5 Hz)	126.0
	ζ		158.4

	C=O		170.7
	NMe	3.07 (s)	30.6
	α	4.53 (m)	57.4
	βa	2.91 (d, *J* = 11.9 Hz)	35.7
	βb	3.03 (d, *J* = 11.9 Hz)	
	γ		127.5
	δa	6.53 (d, *J* = 6.7 Hz)	121.6
Tyr6	δb	4.31 (s)	113.2
	εa	6.76 (ov)	116.1
	εb		153.3
	ζ		143.1
	C=O		168.3
	NMe	2.65 (s)	29.5
